# Chip-scale high Q-factor glassblown microspherical shells for magnetic sensing

**DOI:** 10.1063/1.5030460

**Published:** 2018-06-14

**Authors:** Eugene Freeman, Cheng-Yu Wang, Vedant Sumaria, Steven J. Schiff, Zhiwen Liu, Srinivas Tadigadapa

**Affiliations:** 1 Honeywell International, Aerospace Advanced Technology, Plymouth, MN 55441, USA; 2 School of Electrical Engineering and Computer Science, Pennsylvania State University, University Park, PA 16802, USA; 3 Department of Electrical and Computer Engineering, Northeastern University, Boston, MA 02115, USA; 4 Department of Engineering Science and Mechanics, Center for Neural Engineering, The Pennsylvania State University, University Park, PA 16802, USA; 5 Departments of Neurosurgery and Physics, The Pennsylvania State University, University Park, PA 16802, USA

## Abstract

A whispering gallery mode resonator based magnetometer using chip-scale glass microspherical shells is described. A neodynium micro-magnet is elastically coupled and integrated on top of the microspherical shell structure that enables transduction of the magnetic force experienced by the magnet in external magnetic fields into an optical resonance frequency shift. High quality factor optical microspherical shell resonators with ultra-smooth surfaces have been successfully fabricated and integrated with magnets to achieve Q-factors of greater than 1.1 × 10^7^ and have shown a resonance shift of 1.43 GHz/mT (or 4.0 pm/mT) at 760 nm wavelength. The main mode of action is mechanical deformation of the microbubble with a minor contribution from the photoelastic effect. An experimental limit of detection of 60 nT Hz^−1/2^ at 100 Hz is demonstrated. A theoretical thermorefractive limited detection limit of 52 pT Hz^−1/2^ at 100 Hz is calculated from the experimentally derived sensitivity. The paper describes the mode of action, sensitivity and limit of detection is evaluated for the chip-scale whispering gallery mode magnetometer.

## I. INTRODUCTION

Room temperature, low cost magnetic sensing for bio-magnetic applications are being pursued over a wide range of technologies including atomic magnetometers,^1^ nitrogen-vacancy in diamond magnetometers,^2,3^ and magnetoelectric magnetometers.^4,5^

Recently some efforts have also been made to take advantage of optical resonance methods of sensing to probe magnetic fields.^6–10^ One such strategy is to immerse a fiber based resonator into a magnetic fluid, and then quantify the spectral response while applying an external magnetic field. Magnetic fluids can have large changes in refractive index in response to a magnetic field.^7^ However, ferrofluids can have unacceptably high optical absorption and need to be diluted to balance out refractive index sensitivity in response to magnetic fields and optical losses. The full-width at half-maximum of resonances in most of the fiber-based interferometers, reported thus far, is relatively large, thus hindering a high sensitivity of the magnetic field sensing. Zu et al. demonstrated a sensitivity of 167 pm/mT with a resolution of 60 μT, at low frequencies (DC-100 Hz), with a magneto-optical fiber.^11^ Recently, Amili et al., demonstrated a silicon micro-ring with a magnetic fluid cladding with a sensitivity of 16.8 pm/mT at DC magnetic fields; however the quality factor (Q-factor) of the device suffered significantly after application of the magnetic fluid.^8^ Luo et al. demonstrated an impressive 1918 pm/mT using optical fiber and magnet fluid, but did not report the frequency regime of the measurement nor a limit of detection.^9^

Whispering gallery mode (WGM) optical resonators have demonstrated exceptional sensitivity to mechanical deformation, with shot noise limited displacement sensitivities of 10^−19^ m Hz^−1/2^ experimentally measured at room temperature.^12^ Recently, Forstner et al.^10^ used a toroidal WGM resonator with a Terfenol-D bead to experimentally demonstrate an optomechanical method of magnetic sensing with 400 nT Hz^−1/2^ limit of detection at mechanical resonance of approximately 10 MHz, which is too fast to detect biomagnetic signals. More recent work by Forstner et al.,^13^ improved the optomechanical coupling to the magnetostrictive element, resulting in low frequency detection down to 150 nT Hz^−1/2^ at 2 Hz using frequency and 150 pT Hz^−1/2^ at mechanical resonance in the MHz range.

In this work we leverage the emerging field of optomechanical sensing, for low frequency magnetometer applications. We use recently developed chip-scale glass microspherical shells as the high quality factor WGM resonator platform^14^ and integrate with a micro-magnet to couple magnetic force into mechanical deformation of the microbubble. The integration method described in this work does not add losses and maintains the ultra high > 10^7^ Q-factor of the resonator. The sensitivity and limit of detection is experimentally evaluated for the device. The magnetic force is analytically estimated and the mechanical deformation is modeled using COMSOL to correlate the radial deformation of the microbubble and the mechanically induced stress-based photoelastic component. Finally, we estimate the thermorefractive limited magnetic limit of detection for a fused silica microbubble.

## II. FABRICATION AND TESTING

The chip-scale glassblowing process was first pioneered by Eklund and Shkel^15^ for mechanical resonance applications. The process was modified and adapted for optical resonance as published in our earlier work,^14^ which provides more detail on the fabrication process and WGM resonance achievable using these chip-scale microbubbles.

First, 380 μm diameter circles are patterned using positive photoresist on a silicon substrate. A deep reactive ion etch process is used to etch 250 μm of silicon. The photoresist is stripped away with RemoverPG at 70 °C for 15 minutes, followed by a piranha clean (H_2_SO_4_:(30%)H_2_O_2_ 3:1) to remove any remaining photoresist. Next, the etched silicon is anodically bonded to a 100 μm thick Pyrex^®^ 7740 wafer at 133 kPa (∼1000 Torr), trapping N_2_ inside the cavity. The wafer is diced and a 10 minute 49% HF etch is done to thin the borosilicate glass to 37.5 μm thickness. This etching step allows one to control the final shell thickness. The etched die is placed in a 13.3 kPa (∼100 Torr) vacuum and heated on a silicon nitride ceramic heater to 775 °C for 30 seconds and allowed to rapidly cool to approximately 550 °C, followed by a gradual reduction in temperature to prevent thermally shocking the thin glass microbubbles. During the glassblowing process, the borosilicate softens and begins to expand until the pressure inside the microbubble equals the pressure outside the microbubble. The final shell thickness at the equator is approximately 1.8 μm (measured by scanning electron microscopy) after glassblowing.

**FIG. 1.**
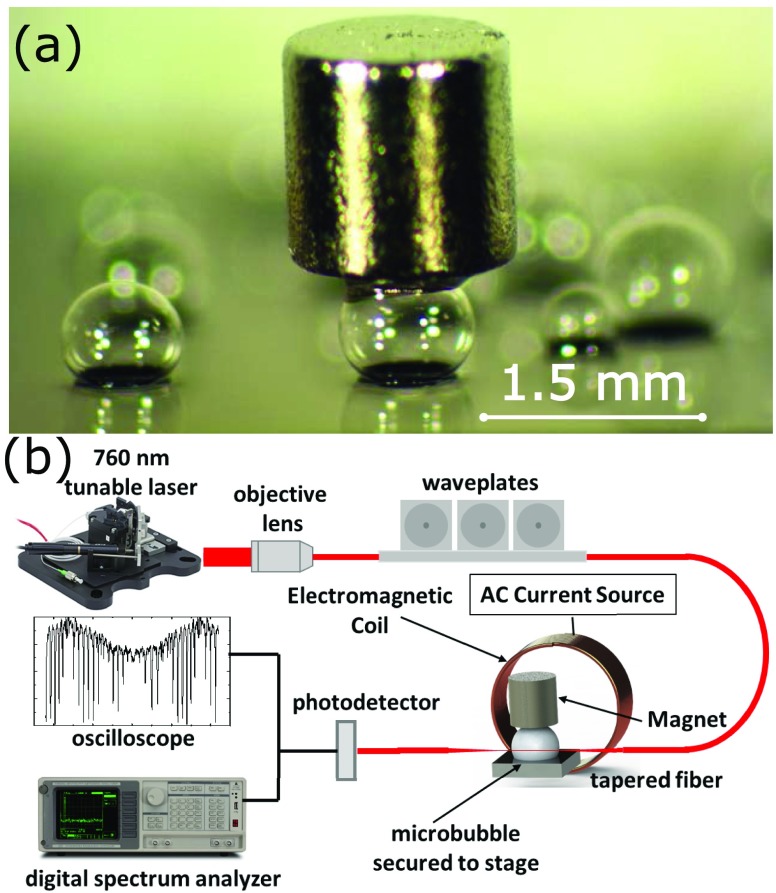
(a) Microscope image of the N48 micromagnet epoxied to the top of the microbubble. (b) Test setup and electromagnetic coil for applying a controlled magnetic field.

By bonding at 1000 Torr and glassblowing at 100 Torr we can create a larger pressure differential that would be possible if both steps were done at atmospheric pressure. This enables larger near spherical microbubbles where the equatorial plane is located above the silicon substrate plane (critical for WGM light confinement with minimal coupling loss to the substrate) without using a complex silicon wafer bonding process, to increase the gas cavity volume, as was demonstrated by others.^15^ Finally, Devcon 5 minute epoxy is used to carefully attach a N48 grade 1.5 mm diameter and 1.5 mm long neodymium magnet to the top of the microbubble, perpendicular to the sample plane, as seen in Fig. 1(a). It is critical to keep the epoxy from contaminating the equatorial plane of the microbubble, which is crucial for the optical resonance. Earlier proof of concept experimental and simulation work showed that epoxying the micromagnet parallel to the sample plane resulted in poor sensitivities. COMSOL simulations shows that the in-plane magnetic forces only caused a smearing of the microsphere, yielding a negligible change in the radius of the microsphere and subsequently a small change in optical path length.^16^ The device is therefore a vector magnetic field sensor which is sensitive to the out-of-plane magnetic field component.

The experimental setup used to apply the magnetic field and probe the optical resonance is illustrated in Fig. 1(b). A 25 mm diameter, 17 mm long, 23 turn coil is wound and the device is placed 8 mm from the edge of the coil. Light from a 760 nm tunable laser (Thorlabs, TLK-L780m) is evanescently coupled to the microbubble through a tapered fiber. A set of waveplates are used to control the polarization to optimize the coupling efficiency. The transmission is measured using a photodiode (Thorlabs, DET36A). The magnetic field produced by the coil is calibrated using a commercial magnetometer (Lakeshore 475, HMMA-2504-VR). To test the magnetic sensitivity, the magnetic field is swept from 0 μT to +630 μT and then back to 0 μT, then to -630 μT and again back to 0 μT. The shift in resonance frequency is monitored by an oscilloscope while the laser frequency is swept. The sample is secured to the stage to minimize motion perturbation and potentially de-coupling from the fiber. To test the limit of detection and noise characteristics of the setup, the laser is held to the steepest portion of a WGM resonance peak and a dynamic spectrum analyzer (Stanford Research SR760) is used observe the signal to noise floor while applying a known signal.

## III. RESULTS AND DISCUSSION

### A. High Q-factor WGM resonance

The glassblowing fabrication method used in this work relies on the reflow of the borosilicate glass enabling an extremely smooth surface. This process is similar in principle to other glass reflow methods that have been used to demonstrate near ideal Q-factors of 8 × 10^9^ in reflown fused silica microspheres.^17^

**FIG. 2.**
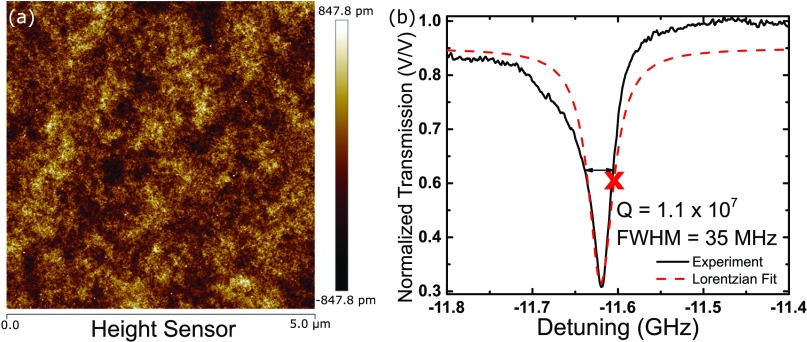
(a) 5 μm x 5 μm section on the top of the microbubble measured by AFM with 0.243 nm RMS roughness and an autocorrelation length of 108 nm. (b) WGM resonance of a glassblown microbubble with 1.1 × 10^7^ Q-factor. The red X marks the approximate location of the steepest portion of the resonance, which is used for testing the limit of detection.

Atomic force microscopy (AFM) is used to measure the roughness on the top of the microbubble. Fig. 2(a) shows the topography of a 5 μm x 5 μm section near the top of the microbubble. The RMS roughness is measured to be 0.243 nm and the correlation length is 108 nm. This is very close to the 0.23 nm roughness reported for glassblown fused silica microbubbles^18^ used for high mechanical Q-factor gyroscopes and 0.33 nm RMS roughness reported for ultra smooth glass surfaces.^19^

The Q-factor (*Q*_*tot*_) of an optical WGM resonator can be summarized by Eq. 1

$$Q_{tot}^{-1}=Q_{rad}^{-1}+Q_{bulk}^{-1}+Q_{s.s}^{-1}+Q_{cont}^{-1}$$ (1)

where *Q*_*rad*_ is due to the radiative (curvature) losses, *Q*_*bulk*_ represents material attenuation losses, *Q*_*s*.*s*_ is due to light scattering from material roughness, and *Q*_*cont*_ represents losses due to contaminants producing surface scattering.^20^ For all calculations the resonant wavelength is approximated as 760 nm, the effective index of refraction is 1.467, and the diameter of the microbubble is measured to be 880 μm. Radiative loss exponentially decreases with increasing resonant optical cavity diameter and can be usually ignored^21^ when *D*/*λ* > 15. In this work the *D*/*λ* is 1158 and can be safely ignored.

The surface scattering can be calculated^17^ using Eq. 2

$$Q_{s.s}=\frac{\lambda^{2}D}{2\pi^{2}\sigma^{2}B}$$ (2)

where *D* is the diameter of the microbubble, *λ* is the resonance wavelength, *σ* is the RMS roughness and *B* is the correlation length. Using the AFM data a *Q*_*s*.*s*_ of 4.04 × 10^9^ is calculated.

Some contaminants can be modeled, for example adsorption of water on the microbubble surface has also been shown to play a role in scattering of high Q-factor WGM resonators. The Q-factor due to adsorbed water scattering, $Q_{w}$, can be calculated^22^ by Eq. 3

$$Q_{w}=\sqrt{\frac{\pi D}{8n^{3}\lambda }}{\left(\delta \beta_{w}\right)}^{-1}$$ (3)

where *δ* is the thickness of the water layer and $\beta_{w}$ is the absorption coefficient of the water. *δ* can be assumed to be 0.2 nm and the absorption coefficient for water at 760 nm is calculated to be 2.52 m^−1^ based off extinction coefficient measurements.^23^ $Q_{w}$ is calculated to be 2.38 × 10^10^.

The material losses due to the attenuation of the laser is calculated^17^ using Eq. 4,

$$Q_{bulk}=\frac{2\pi n_{r}}{\lambda \alpha }$$ (4)

where *n*_*r*_ is the effective index of refraction, and *α* is the attenuation coefficient. Specifications for borosilicate optical fibers^24^ report the attenuation as 520 dB/km (approximately 0.12 m^−1^). The *Q*_*bulk*_ is calculated to be 1.0 × 10^8^ and is the main theoretical limiter of the Q-factor in this work. *Q*_*tot*_ is therefore 9.8 × 10^7^. Other non-idealities such as dust and dirt on the microbubbles can further reduce the Q-factor. The device fabricated for this experiment has an exceptional Q-factor of 1.1 × 10^7^, with a full-width at half-maximum of 35 MHz as seen in Fig. 2(b). Previous studies^14^ yielded borosilicate chip-scale WGM devices with similar Q-factors up to 5.2 × 10^7^, close to the theoretical absorption limit of 9.8 × 10^8^ calculated in this section for borosilicate WGM resonators.

### B. Magnetic sensitivity and mode of operation

**FIG. 3.**
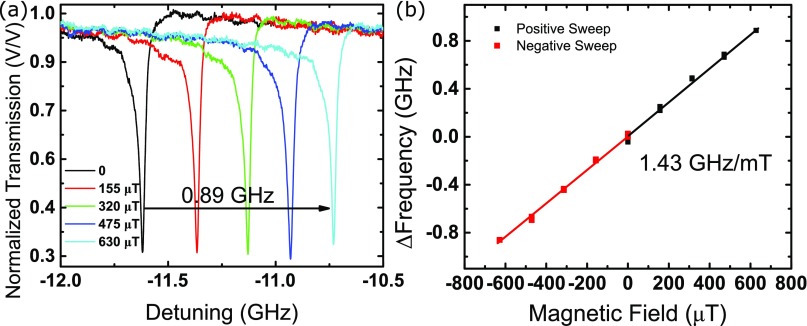
(a) Sensitivity of the WGM is evaluated by tracking the frequency shift. (b) Frequency shift is linear with a sensitivity of 1.43 GHz/mT and shows little hysteresis.

Fig. 3(a) shows the magnetometer sensitivity measured by applying a calibrated magnetic field through the electromagnetic coil and the shift in WGM resonance frequency is tracked. The magnetically induced response is plotted in Fig. 3(b), where a linear fit is used to determine a sensitivity of 1.43 GHz/mT. The device shows little hysteresis and the frequency shift switches from a red-shift (negative Δ*f*) to a blue-shift (positive Δ*f*) when the magnetic field direction goes from negative to positive. This confirms that the phenomenon is due to the magnetic forces and not heat from the coil, which would have caused only a red shift. The observed hysteresis is within the drift of the laser system that has been normally observed over the testing time.

The change in the optical path length at the equatorial plane due to the applied magnetic field (*B*) induced mechanical deformation results in a shift in the resonance frequency given by Eq. 5 where *f* is the resonance frequency, *n*_*r*_ is the effective refractive index and *r* is the optical cavity radius, *C* is the photoelastic constant, and *σ* is the stress.

$$-\frac{\Delta f}{f\Delta B}=\frac{\Delta n_{r}}{n_{r}\Delta B}+\frac{\Delta r}{r\Delta B}=\frac{C}{n_{r}}\frac{\sigma }{\Delta B}+\frac{\Delta r}{r\Delta B}$$ (5)

The mechanical force induced by the magnetic field can be analytically estimated using a formula developed by Janssen et al. for orthogonal cuboid magnets.^25^ The coil in this setup is estimated as a permanent magnet with a pseudo magnetic field strength of $B_{coil}=\frac{I_{c}N_{r}\mu_{0}}{l_{c}}$, where *I*_*c*_ is the current in the coil, *l*_*c*_ is the length of the coil, *N*_*r*_ is the number of turns, *μ*_0_ is the permittivity of free space. The permanent cylindrical magnet is modeled as a cuboid with a matching volume and magnetization of the neodymium N48 micro-magnet. The resulting force^16^ is 226 μN/mT, mostly in the z-axis (orthogonal to the sample plane). Using these forces as an input to a COMSOL model the mechanical deformation and radial stresses are calculated. By comparing the original and the deformed structure in COMSOL the Δ*R* is simulated to be 0.324 nm for a 1 mT magnetic field. The radial stress is averaged over the equator and determined to be 1.70 × 10^−2^ MPa for a 1 mT magnetic field.

Using Eq. 5, with a photoelastic constant^26^ of 4 × 10^−12^ Pa^−1^ the resonance frequency shift can be calculated from the Δ*R* and *σ* determined from the finite element model. The modeled sensitivity is 0.32 GHz/mT, with 0.29 GHz/mT contribution coming from the mechanical deformation and 0.03 GHz/mT from the photoelastic effect. The modeled sensitivity is smaller than the experimentally observed sensitivity, possible due to the fact that the COMSOL model uses a uniform shell thickness, where as in reality the shell thickness thins out at the top of the microbubble. Therefore the finite element simulation would under estimate the true deformation induced in the microbubble.

### C. Limit of detection

**FIG. 4.**
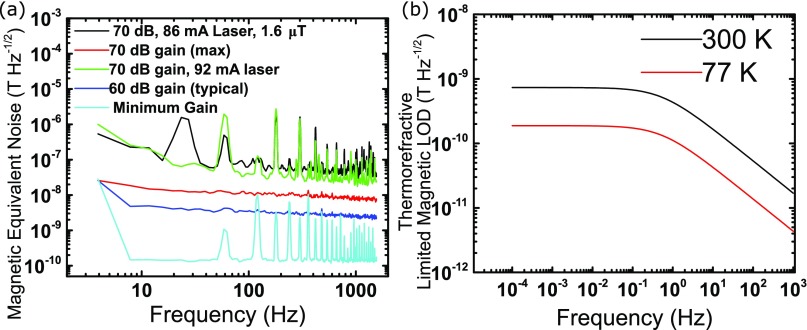
(a) Magnetic equivalent noise from the WGM magnetometer (black line), laser (green line), photodiode at max gain (red line), typical gain (dark blue line) and minimal gain (light blue line). LOD of the device is 60 nT Hz^−1/2^ at 100 Hz (dashed black line), which is limited by laser noise. In an ideal situation the ultimate limit of this setup is set by the photodetector noise at 150 pT Hz^−1/2^. (b) The thermorefractive magnetic equivalent limit of detection for 300 K and 77 K are 52 pT Hz^−1/2^ and 14 pT Hz^−1/2^ respectively.

To test for the limit of detection the laser is detuned by approximately -11.6 GHz to operate the device at the steepest slope region of the WGM resonance as shown by the “X” label in Fig. 2(b). While applying a known 1.6 μT signal at 25 Hz, the response is monitored on the digital spectrum analyzer and a limit of detection of 60 nT Hz^−1/2^ is measured at 100 Hz as seen in Fig. 4.

The dominant noise is evaluated by measuring the noise of the laser without any coupling to the WGM. The green line in Fig. 4 shows that the laser system or the photodetector is the dominant source of noise, not the WGM magnetometer. Next, the photodetector noise is evaluated at maximum gain, typical gain, and minimum gain. It becomes evident that the photodetector has a lower noise floor than the laser. As expected, there is more dark current in the photodetector as the gain increases, producing more noise. A photodetector operating off a DC battery could offer even lower noise, pushing the noise floor lower. Operating at minimum gain on the photodiode and assuming a stable laser and environmental (acoustic and magnetic) noise compensation by common mode rejection (discussed in the next paragraph) on the WGM the limit of detection of this setup can be as low as 150 pT Hz^−1/2^.

Finally we perform a theoretical prediction of the fundamental magnetic limit of detection for a proposed fused silica based microbubble, with low material loss, with an integrated micro-magnet. Several techniques are available to measure optical frequency shifts with high sensitivity which can mitigate the laser noise. For example the balanced homodyne spectroscopy^27^ or by propagating an orthogonal polarization and comparing to the signal beam.^12^ At room temperature thermorefractive noise will likely be the dominating noise source.^28^ Glassblown fused silica microbubbles have been previously demonstrated by other authors for acoustic resonance phenomenon.^29^ It is likely that very high Q-factor fused silica WGM optical resonators are achievable using this process, owing to the lower optical absorption coefficient for fused silica compared to borofloat. We therefore perform the thermorefractive analysis assuming the parameters of a fused silica microbubble of identical dimensions to the borosilicate microbubble fabricated in this work. Furthermore, since the Young’s modulus of fused silica is only slightly higher than borofloat (72 GPa versus 64 GPa) we assume the a similar magnetic sensitivity of 1.43 GHz/mT can be achieved. The thermorefractive noise,^28^ in units of Hz^−1/2^, is given by ${\left(S_{\delta f/f}\right)}^{1/2}=\sqrt{\alpha_{n}^{2}S_{T}\left(\Omega \right)}$, where *α*_*n*_ is the thermorefractive coefficient of the resonator host material, *S*_*T*_(Ω) is given by Eq. 6.

$$S_{T}\left(\Omega \right)=\frac{k_{B}T^{2}}{V_{eff}}\frac{r^{2}}{12\kappa }{\left(1+{\left(\frac{r^{2}\rho C\Omega }{3^{5/2}\kappa }\right)}^{3/2}+\frac{1}{6}{\left(\frac{r^{2}\rho C\Omega }{8l^{1/3}\kappa }\right)}^{2}\right)}^{-1}$$ (6)

where *k*_*B*_ is Boltzmann’s constant, *T* is the temperature, *V*_*eff*_ is the effective mode volume, *κ* is the thermal conductivity, *ρ* is the density, *r* is the resonator radius, *l* is the orbital mode number, and Ω is the noise frequency. The derivation^30^ and constants for fused silica^28^ can be found elsewhere. Using the experimentally derived sensitivity of 1.43 GHz/mT and a approximate resonant frequency of 394 THz (∼ 760 nm) the magnetic limit of detection is plotted in Fig. 4(b) for 300 K and 77 K. The thermorefractive magnetic equivalent limit of detection for 300 K and 77 K are 52 pT Hz^−1/2^ and 14 pT Hz^−1/2^ respectively.

## IV. CONCLUSION

In conclusion, a 1.1 × 10^7^ Q-factor chip-scale whispering gallery mode magnetometer was experimentally demonstrated with a sensitivity of 1.43 GHz/mT. The phenomenon is determined to be mostly due to mechanical deformation of the radius of the borosilicate microbubble with a small contribution from the photoelastic effect. An experimental limit of detection of 60 nT Hz^−1/2^ was measured for our current setup and determined to be dominated by laser noise. A thermorefractive analysis for a fused silica microbubble predicts an ultimate limit of detection of 52 pT Hz^−1/2^ at 100 Hz is possible at room temperature.
